# Smoke exposure, hemoglobin levels and the prevalence of anemia: a cross-sectional study in urban informal settlement in Southern Ghana

**DOI:** 10.1186/s12889-024-18304-4

**Published:** 2024-03-19

**Authors:** Cyril Appiah-Dwomoh, Prudence Tettey, Enoch Akyeampong, Prince Amegbor, Gabriel Okello, Paul K. Botwe, Reginald Quansah

**Affiliations:** 1 School of Public Health, University of Ghana, P.O. Box LG 30, Legon, Accra Ghana; 2 School of Global Public Health, New York University, New York, USA; 3 Institute for Sustainability Leadership, University of Cambridge, Cambridge, UK; 4 African Centre for Clean Air, Kampala, Uganda

**Keywords:** Anemia, Ambient air pollution, Informal settlement, Household air pollution, Smoke exposure

## Abstract

**Background:**

In sub-Saharan African cities, more than half of the population lives in informal settlements. These settlements are close to smoky dumpsites, industrial plants, and polluted roads. Furthermore, polluting fuels remain their primary sources of energy for cooking and heating. Despite evidence linking smoke and its components to anaemia, none of these studies were conducted on populations living in urban informal settlements. This study investigated the risks of anemia/mean Haemoglobin (HB) levels in an informal settlement in Accra, Ghana. Exposure to smoke was examined across various sources, encompassing residences, neighborhoods, and workplaces.

**Methods:**

The study was a facility-based cross-sectional design among residents at Chorkor, an informal settlement in the Greater Accra region of Ghana. A questionnaire was administered at a community hospital during an interview to gather data on sources of smoke exposure in the household, in the neighbourhood, and in the workplace. A phlebotomist collected blood samples from the participants after the interview to assess their anaemia status.

**Results:**

The population (*n* = 320) had a high prevalence of anemia, with 49.1% of people fitting the WHO’s definition of anemia, while the average HB level was 12.6 ± 2.1 g/dL. Anemia was associated with the number of different types of waste burnt simultaneously [(1 or 2: prevalence ratio (PR): 95% confidence interval (CI), 1.14, 0.99–1.28: 3+: 1.16, 1.01–1.63, p-for-trend = 0.0082)], fuel stacking [(mixed stacking: 1.27, 1.07–1.20: dirty stacking:1.65, 1.19–2.25, p-for-trend = 0.0062)], and involvement in fish smoking (1.22, 0.99–1.06). However, the lower limit of the CIs for number of different forms of garbage burned simultaneously and engagement in fish smoking included unity. Reduced mean HB levels were associated with the number of different types of waste burnt simultaneously [(1 or 2: regression coefficient (β): 95% confidence interval (CI), -0.01, -0.97- -0.99: 3+: -0.14, -0.77- -0.05)], current smoker [(yes, almost daily: -1.40, -2.01- -0.79: yes, at least once a month: -1.14, -1.79- -0.48)], Second-Hand-Smoking (SHS) (yes, almost daily: -0.77, -1.30- -0.21), fuel stacking [(mixed stacking-0.93, -1.33–0.21: dirty stacking-1.04, -1.60- -0.48)], any smoke exposure indicator in the neighbourhood (-0.84, -1.43- -0.25), living close to a major road (-0.62, -1.09- -0.49), and fish smoking (-0.41,-0.93- -0.12).

**Conclusion:**

Although the cross-sectional design precludes causality, smoke exposure was associated with mean HB levels and anaemia among populations living in informal settlements.

## Introduction

Anemia is a condition in which theHB levels are below the Lower limit of Hb threshold per sex (≤ 13.0 g/dL for men and ≤ 12.0 g/dL for women) [1]. The prevalence of anemia is 39.8% worldwide, but it is higher in Africa and Asia, and significantly higher in poor resource settings in these regions [2]. In urban informal settlements in Asia, the prevalence of anemia ranges from 38.7 to 50.7% in children and adolescents, from 60 to 68% in women of reproductive age, and about 60.6% in the elderly [2–5]. However, there is a dearth of information regarding the prevalence of anemia in urban slums in Africa, particularly in sub-Saharan Africa.

Anemia is caused in approximately 75% of cases by nutritional inadequacies, parasite infections, infectious illnesses, maternal blood loss, and inflammation [6–9]. Another factor associated with anemia among children and adults is smoke exposure from biomass combustion [3]. Chronic exposure to smoke particulate matter (PM) is known to disrupt iron homeostasis by inducing local and systemic inflammatory reactions via an oxidative stress pathway, especially in people with chronic diseases like obesity, chronic kidney disease, and autoimmune conditions [9, 10]. Carbon monoxide (CO) combines with hemoglobin (Hb) in the blood to form carboxyhemoglobin (COHb), which significantly reduces the amount of hemoglobin available for oxygen transport [3, 5]. In addition, COHb can increase the affinity of oxygen for HB, making it more difficult for oxygen to enter tissues and increasing the risk of arterial hypoxaemia [5]. Polycyclic aromatic hydrocarbons (PAH) can also alter the morphology of red blood cells and reduce their oxygen carrying capacity, eventually leading to cell lysis [3, 5].

Even though some research has found a relationship between smoke and its components and the risk of anemia, the evidence is not sufficient and, in most cases, conflicting. Armo-Annor et al. [6] compared the risk of anaemia among women fish smokers with women non-fish smokers in rural Ghana. The authors observed an increased risk of anaemia among fish smokers. This finding was corroborated in large studies among children in India [9, 10] and in sub-Saharan Africa [11]. But among children in urban Swaziland [7], this finding was not confirmed. Around 75% of Africa’s urban population, particularly in Sub-Saharan Africa, lives in informal settlements [8]. These settlements are located close to industrial facilities, smoke-filled dumpsites, and polluted roadways. Solid fuels are also used extensively in these settings. Residents in these communities are exposed to high concentrations of a different mix of pollutants in smoke. The composition of smoke in these settlements may vary widely from those in affluent locations in the urban areas. As far as we are aware, no study has examined the relationship between the numerous smoke exposure sources found in slum areas and the risk of anaemia/HB levels in Low- and Middle-Income Countries (LMICs). This study investigated the prevalence of anaemia/HB levels in an informal settlement in Accra, Ghana. Smoke exposure was looked at from a variety of sources, including those in homes, neighbourhoods, and places of work.

## Materials and methods

### Study design, setting and participants

This facility-based cross-sectional study was conducted at Chorkor (from April to July 2020), an informal settlement in Ghana’s Greater Accra region in the Ablekuma South constituency. Chorkor is a densely populated region where Ga is the primary language. Chorkor’s population is around 344,627 at the time of the 2021 census, with an annual growth rate of 6.0%. The community’s main source of income is fishing. On the other hand, some engage in commercial driving and street vending of both cooked and uncooked food. There is a community hospital at the heart of the community that offers residents 24-hour medical services. This study used a single population proportion formula [8] to arrive at a sample size of 320 [(95% confidence interval of 1.96, margin of error = 0.05, and prevalence of anemia = 41.2% [10]]. The study was conducted at the height of the COVID-19 pandemic in Ghana (when hospital attendance was low). Thus, a convenient sample technique was used. The inclusion criteria for participation in the study were being an adult (i.e., > 18 years old), seeking a laboratory service for a full blood count test, and having lived in the community for at least 6 months. Patients with chronic diseases such as hypertension, diabetes, chronic kidney disease, chronic liver disease, HIV, and those who were critically ill were excluded from participating. Prior to recruitment, hospital records were used to verify patient status.

### Data collection

Trained field workers interviewed participants with a slightly modified questionnaire used in a World Health Organization Urban Health Initiative project in Accra by us [12]. The questionnaire included questions related to personal information such as age, sex, marital status, level of education, sanitation, household income, use of anti-helminthic and rapid diagnostic test, and occupation. There were also questions related to sources of smoke/fumes exposure such as garbage burning, primary/secondary fuel use, exhaust fumes in dwelling, use of mosquito coil, cigarette smoking, ventilation; and how often participants are exposed to the smoke/fumes. Also included were questions related to sources of smoke in the neighborhood such as closeness to major roads, exposure to smoke from dumpsite/garbage heap, and smoke from neighbor’s compound. The questionnaire also captured smoke encountered at the workplace such as exhaust fumes and fish smoking activities. The questionnaires were completed at the community hospital which is at the heart of the community. Each participant spent on average 15 min to complete the questionnaire. Just after the completion of the questionnaire, trained phlebotomists took blood from participants. The participant was asked to flex the arm and a tourniquet was applied to the upper arm. The mid cubital vein was located, and the puncture site cleaned with 70% alcohol swabs. Venipuncture was made with the needle of the syringe at an angle of approximately 45° to the puncture site. Two millimeter of blood was steadily collected into Ethylenediaminetetraacetic acid (EDTA) tubes. The tourniquet was released, and the needle withdrawn from the vein. A ball of cotton wool was immediately placed at the puncture site. The blood was put into EDTA tubes and thoroughly mixed with the EDTA solution. The blood samples were transferred to the laboratory and through the colorimetric method, the mindray BC 20 was used to estimate the total hemoglobin concentration of the sampled blood. The value of the hemoglobin level was compared to the value of the hematocrit level (which is approximately 3 times the value of the hemoglobin level) to ensure accuracy of the results obtained. Venipuncture was preferred to a finger prick during the blood sample collection because blood samples obtained by finger prick are usually more likely to either contain more blood cells than plasma or more plasma than blood cells as there is a possibility of the blood easily clotting. The milking of the patient to get the required amount of blood could be inconvenient and painful when a finger prick is applied. It is standard laboratory practice to always run commercially supplied standards concurrently with a known sample as a quality check. We used BC-3D Hematology Control-Tri-level commercial standard in the lab.

### Outcome of interest

The outcome of interest was anemia, defined as a blood HB concentration of 12.0 g/dL in women and 13.0 g/dL in men by the World Health Organization [12]. The level of HB is treated as a continuous variable.

### Determinant of interest

The primary determinant of interest included indicators of smoke exposure at home, in the neighborhood, and at the workplace derived from the questionnaire. Smoke exposure indicators at home were any smoke exposure at home (yes or no), residential garbage burning (yes or no), the number of different types of waste openly burned by the household (I do not burn waste, I burn 1 or 2 types of waste, I burn more than 3 different types of waste), how often waste is openly burned at home (do not burn waste, almost daily, at least once a month), current smoker (never, yes almost daily, yes at least once a month), SHS (never, yes almost daily, yes at least once a month), burning of mosquito coil (yes vs. no), fuel stacking (clean stacking, mixed stacking, dirty stacking), frequency of cooking (1–2 times a month, 1–2 times a week, almost daily), duration of cooking (less than 4 h vs. > 4 h), location of cooking (outdoor, in an open area/under a shed, enclosed space, combined area), time spent in the kitchen (at most a quarter of the time, half of the time). Smoke exposure indicator in the neighborhood included how frequently neighbors openly burnt waste (never, almost daily, at least once a month), exposure to smoke from a nearby dumpsite/garbage heap (never, almost daily, at least once a month), and living near a major road (yes vs. no); and smoke exposure indicator at work also included any exposure to smoke at work, exposure to smoke, exposure to exhaust fumes at work (never, almost daily, at least once a month), and involvement in fish smoking (yes vs., no).

### Confounders

Potential confounders controlled for in our analysis were age (< 30 yrs vs. ≥30 yrs), sex (male vs. female), household monthly income (< 1000 vs. ≥ 1000 Ghana Cedis), Body Mass Index (BMI) (normal vs. overweight/obese), number of people living in a household (< 3 vs. ≥ 3), use of anti-helminthics (yes vs. no), and results for Rapid Diagnostic Test (RDT) for malaria parasites (positive vs. negative). The selection of these variables was informed by literature [4, 11, 16–21] and their significant association with anaemia prevalence/mean HB level and any smoke exposure (derived from our exposure indicators at home, in the neighbourhood, and at the workplace: see Table 1) at *P* < 0.05.

### Statistical analysis

We computed means and standard deviation for age (continuous) and mean HB levels. We also computed proportions or percentages for our nominal (e.g., sex, marital status, religion) ordinal (e.g., last time you took anti-helminthic, body mass index) and interval variables (e.g., age, household income). First, we applied generalized linear models (SAS PROC GENMOD) with binomial distribution and log link to assess the potential association between smoke/fumes exposure indicators and anemia. Prevalence ratio (PR) was the effect measure. We further applied a multivariable linear regression for the association between smoke/fumes exposure indicators and hemoglobin levels. The analysis was performed with the SAS statistical software package (SAS, version 9.4, SAS Institute, Cary, NC). To assess the model fitness for the logit scale, the Hosmer-Lemeshow goodness-of-fit test was used which was not significant (P-value > 0.05) indicating that the fit model was matched the distribution/ the data generation price of the data. The Area under the curve was also estimated to be 0.78 which is within the acceptable range for model discriminating performance. For the linear model, the normality of the residuals was explored using the histogram, q-q plot and p-p plot. Also, Shapiro-Wilk and Shapiro-Francia tests for normality were also used to formally test the normality of the residual and the P-values were greater than 0.05 which show that the residuals follow the Gaussian distribution. The robust standard errors were used instead of the regular standard errors to overcome any issue of heteroskedasticity of the variance. Multicollinearity was not an issue as all the model covariates had a variance inflation factor of less than 10 (Rage of VIF from the model was 1.5–4.7) [12].

## Results

### Participant characteristics

**Fig. 1:**
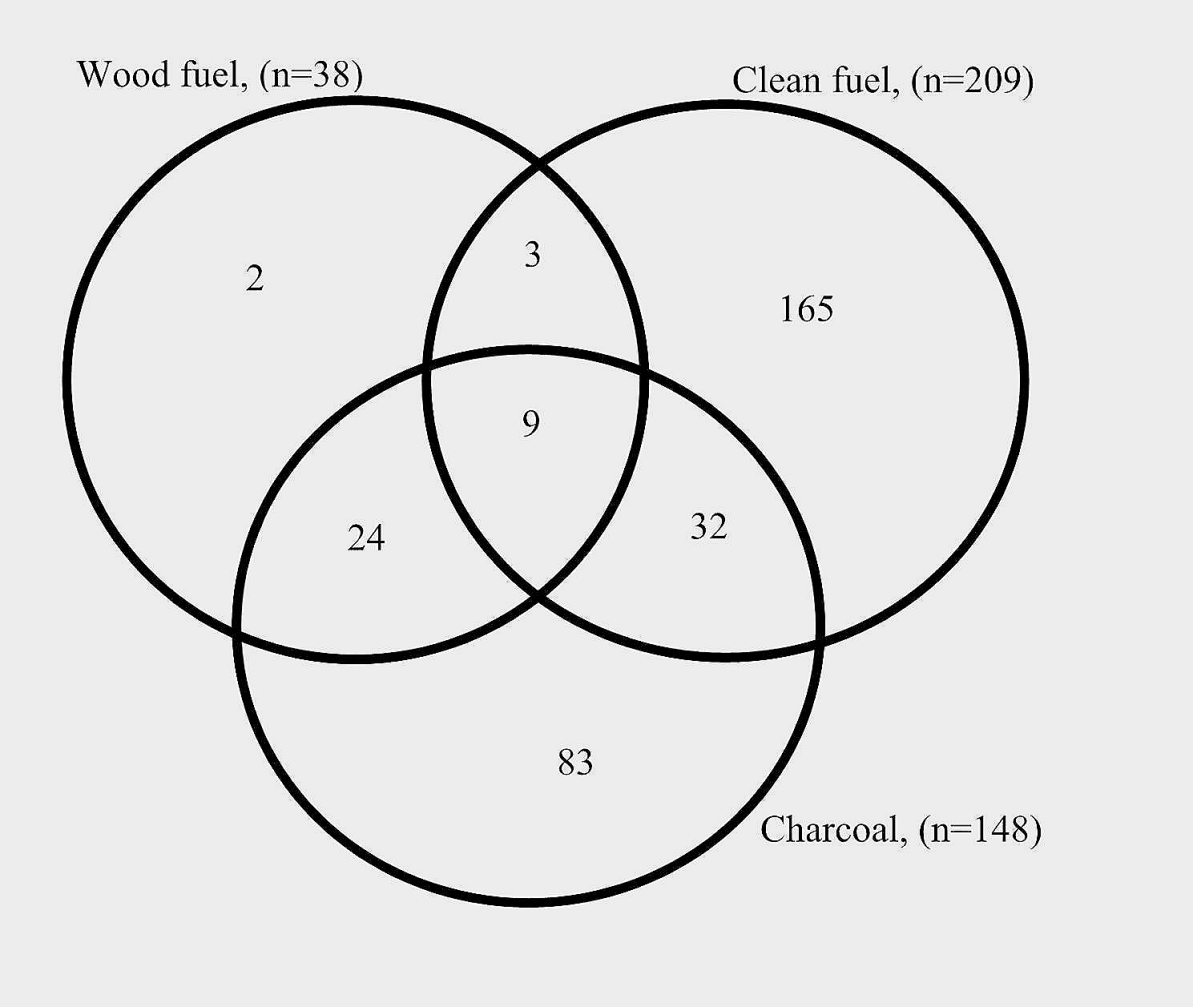
Choice of cooking fuel in participants’ households (*n* = 320)

Most of the participants were below 30 years, 50.9% were males, 62.5% were either married or cohabiting, 63.1% belong to the Ga tribe, 48.8% were in the fisheries or selling or general merchant business, 53.8% had up to pre-primary/primary/Junior High School (JHS)/Senior Secondary School (SSS) education, 45.6% were obese, 58.4% lived in a household with improved sanitation facility and 87.2% had access to improved source of drinking water. 42.5% had taken anti-helminthic medication within the last 3 months or tested negative on Rapid Diagnostic Test (RDT).

(Table 2) Out of 320 participants surveyed, about 52% exclusively used clean fuel, as against 26% and less than <0.01% who exclusively used charcoal and wood fuel. Only 0.03% of households used all three fuel types (Fig. 1). Households burnt mosquito coil (yes vs no: 43.4% vs 56.6%) or reside close to a major road (48.4% vs. 51.6%) or are involved in fish smoking (22.7% vs. 7.04) or are exposed to smoke from a nearby dumpsite/garbage heap (18.8% vs. 81.3%) or exposed to SHS (50.3% vs. 49.7%), smoke (25% vs. 75%) or are engaged in residential garbage burning (17.5% vs. 82.5%).

**Table 1:** Model associations between smoke exposure indicators and the risk of anemia/hemoglobin levels

Smoke Exposure Indicators	Anemia Risk		Hemoglobin level	
	Crude PR (95%CI)	Adjusted PR (95%CI)	Crude β(95%CI)	Adjusted β (95%CI)
**Any Smoke Exposure**				
No	1.00	1.00	0.00	0.00
Yes	1.01 (0.87–1.17)	0.98 (0.69–1.40)	-0.35 (-0.85-0.15)	-0.56 (-1.19-0.08)
***Smoke Exposure Indicators at home***				
*Any smoke exposure at home*				
No	1.00	1.00	0.00	0.00
Yes	1.01 (0.85–1.21)	1.03 (0.69–1.53)	0.43 (-0.17-1.02)	0.32 (-0.38-1.02)
*Household garbage burning*				
No	1.00	1.00	0.00	**0.00**
Yes	1.02 (0.72–1.42)	1.06 (0.76–1.49)	0.43 (-0.17- 1.02)	**0.36 (-0.20- 0.92)**
*Number of different type of wastes burnt by the household*				
0	1.00	1.00	0.00	0.00
1 or 2	1.15 (1.09–1.13)	**1.14 (0.99–1.28)**	0.73 (-0.04- 1.50)	**-0.01 (-0.97–0.99)**
3+	1.21 (1.00-1.26)	**1.16 (1.01–1.63)**	0.05 (-0.79- 0.90)	-0.14 (-0.77–0.05)
p-trend	0.0068	**0.0082**		
*Do you burn mosquito coil indoors*				
No	1.00	1.00	0.00	0.00
Yes	1.04 (0.91–1.99)	0.98 (0.75–1.28)	0.09 (-0.37- 0.54)	-0.10 (-0.53-0.34)
*Current cigarette smoker*				
Never	1.00	1.00	0.00	0.00
Yes, almost daily	0.79 (0.69–0.91)	0.89 (0.81–0.98)	-1.36 (-2.06- -0.67)	**-1.40 (-2.01- -0.79)**
Yes, at least once in a month	0.63 (0.47–0.84)	0.94 (0.90–0.99)	-1.30 (-1.94- -0.67)	**-1.14 (-1.79- -0.48)**
p-for-trend	0.0002	0.0131		
*Exposed to SHS*				
Never	1.00	1.00	0.00	0.00
Yes, almost daily	0.78 (0.65–0.95)	0.89 (0.64–1.25)	-0.78 (-1.34–0.23)	**-0.77 (-1.30–0.24)**
Yes, at least once in a month	0.61 (0.42–0.90)	0.80 (0.41–1.56)	-0.50 (-1.04-0.04)	-0.34 (-0.85-0.17)
p-for-trend	0.0054	0.5042		
*Fuel stacking*				
Clean stacking	1.00	1.00	0.00	0.00
Mixed stacking	1.31 (1.14–1.49)	1.27 (1.07–1.50)	-0.09 (-0.65- -0.84)	**-0.93 (-1.33- -0.21)**
Dirty stacking	1.71 (1.30–2.23)	1.65 (1.19–2.25)	-0.80 (-1.28- -0.32)	-1.04 (-1.60- -0.48)
p-for-trend	0.0008	0.0062		
*Frequency of cooking*				
1–2 times in a month	1.00	1.00	0.00	0.00
1–3 times in a week	0.89 (0.82–0.96)	0.92 (0.61–1.38)	-1.49 (-2.17- -0.81)	**-1.04 (-1.69- -0.41)**
Almost daily	0.79 (0.67–0.93)	0.96 (0.78–1.17)	-1.61 (-2.30–0.92)	**-1.38 (-2.03- -0.73)**
p-for-trend	0.008	0.6835		
*Average duration of cooking*				
≤ 4 h	1.00	1.00	0.00	0.00
>4 h	1.09 (0.82–1.45)	1.35 (0.66–2.76)	0.71 (-0.52- 1.94)	1.46 (0.29–2.62)
*Location where cooking was done*				
Outdoor, in an open area/under a shed	1.00	1.00	0.00	0.00
Enclosed space	0.79 (0.69–0.90)	0.85 (0.62–1.17)	**-1.43 (-1.91- -0.95)**	**-1.24 (-1.73- -0.75)**
Combined area	0.89 (0.83–0.95)	0.92 (0.79–1.08)	**1.09 (0.36–1.83)**	0.55 (-0.20- 1.29)
p-for-trend	0.0004	0.3123		
*Time spent in the kitchen*				
At most a quarter of the time	1.00	1.00	0.00	0.00
Half of duration	1.03 (0.93–1.14)	0.95 (0.77–1.17)	-0.69 (-1.36- -0.01)	**-0.93 (-1.56- 0.31)**
Whole duration	1.06 (0.87–1.29)	0.90 (0.59–1.36)	0.18 (-0.50- 0.85)	-0.65 (-1.31- 0.01)
p-for-trend	0.6091			
***Smoke Exposure Indicator in the neighborhood***				
*Any smoke exposure indicator in the neighbourhood*				
No	1.00	1.00	0.00	0.00
Yes	0.88 (0.77–1.01)	0.90 (0.64–1.27)	-0.67 (-1.12- -0.22)	**-0.84 (-1.43- -0.25)**
*How often do your neighbors openly burnt waste at home?*				
Neighbors do not burn garbage	1.00	1.00	0.00	0.00
Yes, almost daily	1.21 (1.06–1.39)	**1.23 (1.02–1.60)**	-0.26 (-1.12- 0.60)	-0.17 (-0.99- 0.65)
Yes, at least once in a month	1.10 (1.03–1.18)	**1.15 (0.99–1.26)**	1.48 (1.01–1.96)	1.21 (0.74–1.68)
p-trend	0.0101	0.02071		
*Exposed to smoke from a nearby dumpsite/garbage heap?*				
Never	1.00	1.00	0.00	0.00
Yes, almost daily	0.95 (0.86–1.05)	0.89 (0.62–1.30)	0.82 (-0.37- 2.01)	0.29 (-0.85- 1.43)
Yes, at least once per month	0.90 (0.74–1.11)	0.95 (0.79–1.14)	-0.54 (-1.18- 0.09)	-0.38 (-0.99- 0.22)
p-for-trend	0.2999	0.5491		
*Dwelling close to a major road*				
No	1.00	1.00	0.00	0.00
Yes	1.14 (0.99–1.30)	**1.08 (0.97–1.40)**	-0.67 (-1.12- -0.22)	**-0.62 (-1.09- -0.16)**
***Smoke Exposure Indicator at work***				
*Any Exposure to smoke at work*				
No	1.00	1.00	0.00	0.00
Yes	1.15 (1.00-1.33)	1.16 (0.76–1.74)	-0.13 (-0.67-0.41)	-0.23 (-0.95-0.50)
*Exposed to smoke*				
Never	1.00	1.00	0.00	0.00
Yes, almost daily	1.40 (1.05–1.89)	1.01 (0.71–1.43)	0.20 (-0.37-0.76)	-0.05 (-0.59-0.49)
Yes, at least once in a month	1.19 (1.02–1.37)	1.02 (0.51–2.06)	0.53 (-0.04-1.06)	0.17 (-0.34-0.69)
p-for-trend	0.0386	0.9536		
*Involved in Fish smoking*				
No	1.00	1.00	0.00	0.00
Yes	1.15 (1.00-1.33)	1.22 (0.99–1.40)	-0.13 (-0.67- -0.41)	**-0.41 (-0.93- -0.12)**

**Table 2:** Characteristics of study participants, (*N* = 320)

Participant characteristics	n	(%)
**Age (yrs)**		
< 30	123	38.4
30–40	109	34.1
41+	88	27.5
**Gender**		
Male	163	50.9
Female	157	49.1
**Marital Status**		
^a^Single	120	37.5
Married/cohabiting	200	62.5
**Ethnicity**		
Akan	63	19.7
Ewe	27	8.4
Ga	202	63.1
Hausa	16	5.0
^b^Others	12	3.8
**Occupation**		
Manager/Manageress/Professionals	71	22.2
Fisheries/petty trade	156	48.8
Craftsmanship	60	18.8
Unemployed/pensioner/student	33	10.3
**Highest educational qualification**		
Never attended	38	11.9
Pre-primary/Primary/JHS/SSS	172	53.8
Technical professional certificate/diploma	54	16.9
Bachelor/Postgraduate	56	17.5
**Household monthly income (Ghana Cedis)**		
< 250	40	12.5
250–500	81	25.3
510-1,000	64	20.0
1,100-1,500	75	23.4
1,500-2,000	42	13.1
> 2,000	18	5.6
**Body Max Index**		
Normal Weight	56	17.5
Overweight	118	36.9
Obese	146	45.6
**No. of years lived in the current house (yrs)**		
<5	88	27.5
6–10	80	25.0
11+	152	47.5
**No. of people living in a household**		
< 10	77	24.1
10–20	144	45.0
>20	99	30.9
**Main source of drinking water for household**		
^c^Improved	279	87.2
^d^Unimproved	41	12.8
**Household access to a toilet facility**		
No	133	41.6
Yes	187	58.4
**Type of sanitation facility**		
^e^Unimproved	133	41.6
^f^Improved	187	58.4
**Last time you took anti-helminthic?**		
Within 3 months	136	42.5
More than 3 months ago	90	28.1
Over a year	94	29.4
**RDT for malaria parasite**		
Negative	281	87.8
Positive	39	12.2

Note: JHS is Junior High Secondary, SHS is Senior High Secondary

Note; ^a^Single includes Unmarried, Separated, Widowed or Divorced

^b^Others include: Fafra, Fante, Ga-dangme, Nzema, HB; RDT = Rapid Diagnostic Test)

^c^improved water source refers to piped household water connection system, borehole, protected spring, protected dug well,, public standpipe, rain water collection; ^d^unimproved water source refers to unprotected spring, unprotected dug well, surface water (river, dam, lake, pond, stream, etc.), bottled water, vendor-provided water, tanker truck water)

^e^improved sanitation facility also refers to toilet facilities with sewer, connections, septic tank, pour-flush latrines, ventilated improved pit latrines and pit latrines with slab or covered pit; ^f^unimproved sanitation facility refers hanging latrines, bucket latrines, open defecation, pit latrines with no slabs or open pit); as defined by World Health Organization (2012)

### Prevalence of anemia

Anemia was highly prevalent in the current population with 49.1% meeting the World Health Organization’s definition of anemia; whereas the mean HB level in the population was 12.6 ± 2.1 g/dL. The prevalence of anemia in this population generally increased with age (39.5% at < 30 yrs is higher than 33.1.% at 30–40 yrs., Tau b = 0.0093), higher in females (56.0%) than in males (44%), significantly (Somers D = 0.0800), lower in singles (31.2%) compared to married/cohabitors (66.3%). With respect to ethnicity, anemia prevalence was lower in undefined ethnic group (3.2%) and Hausa (6.4%) compared to the Akan tribes (Somers D = 0.0265). It is also lower in those with no education and technical professional certificate/diploma qualification (14.7%) compared to those with a pre-primary/primary/JHS/SSS qualification (57.3%, Somers D = 0.0203), decrease increased monthly household income (Somers D = 0.0064). The risk of anemia is reduced in those with normal BMI compared to those overweight/obese (Somers D = 0.0076), higher in households with improved source of drinking water compared to those in households with unimproved source of drinking water (Somers D = 0.0537) (Table 3).

**Table 3:** Model association between participants’ characteristics and Hemoglobin status (*N* = 320)

Participants characteristics		Hemoglobin status	
	Total	Anemic	Non-Anemic
**Age (yrs)**			
< 30	123 (38.44)	62 (39.49)	61 (37.42)
30–40	109 (34.06)	52 (33.12)	57 (34.97)
41+	88 (27.390)	43 (27.39)	45 (27.61)
Tau b = 0.0093			
**Sex**			
Male	163 (50.93)	69 (43.95)	94 (57.67)
Female	157 (49.06)	88 (56.05)	69(42.33
Somers D= -0.5605			
**Marital Status**			
Single	120 (37.50	49 (31.21)	71 (43.56)
Married/cohabiting	200 (62.50)	108 (66.26)	92(56.44)
Somers D = 0.0800			
**Ethnicity**			
Akan	63 (19.69)	32 (20.38)	31 (19.02)
Ewe	27 (8.44)	13 (8,28)	14 (8.59)
Ga	202 (63.13	97 (61.78)	105(64.42)
Hausa	16 (5.00)	10 (6.37)	6 (3,68)
Others	12 (3.76)	5 (3.18)	7 (4.29)
Somers D = 0.0265			
**Highest Educational Qualification**			
Never attended	38 (11.9)	14 (8.92	24 (14.72)
Pre-primary/Primary/JHS/SSS	172 (53.8)	90 (57.32)	82 (50.31)
Technical professional certificate/diploma	54 (16.9)	23 (14.65)	31 (19.02)
Bachelor/Postgraduate	56 (17.5)	30 (19.11)	26 (15.95)
Somers D = 0.0203			
**Household Income (In Ghana cedis)**			
< 250	40 (12.50)	18 (11.46)	22 (13.50)
250–500	81 (25.31)	56 (35.57)	25 (15.34)
510-1,000	64 (20.00)	22 (14.01)	42 (25.77)
1,100-1,500	75 (23.44)	51 ()	24 (14.72)
1,500-2,000	42 (13.13)	6 (32.48)	36 (22.09)
> 2,000	18 (5.63)	4 (2.55)	14 (8.59)
Somers D=-0.0064			
**Body Max Index**			
Normal Weight	56(17.50)	25 (15.34)	31 (19.02)
Over weight	118 (36.88)	62 (39.49)	56 (34.36)
Obese	146 (45.63)	70 (44.59)	76 (46.63)
Somers D = 0.0076			
**Main Source of drinking water for household**			
Improved	279 (87.19)	139 (88.54)	140 (85.89)
Unimproved	41 (12.81)	18 (11.46)	23 (18.11)
Somers D=-0.0537			
**Type of sanitation facility**			
Unimproved	133 (41.6)	47 (29.94)	86 (52.76)
Improved	187 (58.4)	110 (70.06)	77 (47.24)
Somers D=-0.2247			
**Last time you took anti-helminthic**			
Within 3 months	136 (42.50)	42 (26.75)	94 (575.67)
More than 3 months ago	90 (28.13)	62 (39.49)	28 (17.18)
Over a year	94 (29.38	53 (33.76)	41 (25.15)
Somers D=-0.1655			
**RDT for malaria parasite**			
Negative	281 (87.81)	127 (80.89)	154 (94.48)
Positive	39 (12.19)	30 (19.11)	9 (5.52)
Somers D=-0.0880			
**Use mosquito spray**			
Yes	148 (46.25	22 (14.01)	59 (3620)
No	172 (53.75)	135 (85.99)	104 (63.80)
Somers D=-0.1834			

### Association of Smoke exposure indicators with anemia

Table 1 displays the association between some exposure indicators and the risk of anemia. Of the smoke exposure indicators measured in the household, the number of different types of waste burnt in the household was significantly associated with the risk of anemia in an exposure-response manner (*p* = 0.0082). The prevalence ratio (PR) of 1 or 2 different types of waste combination burnt and more than 3 different types of waste combination burnt compared to not practicing residential burning were 1.14 (0.99–1.28) and 1.16 (1.01–1.63) respectively. However, any smoke exposure indicator in the household, household garbage burning, frequency of garbage burning at the household, exhaust fumes entering dwelling, burning mosquito coil, current smoker, exposure to SSS were not associated with anemia risk. There was an increasing risk of anemia with fuel stacking options (*p* = 0.0062). That is, using mixed stacking and dirty stacking versus clean fuel. The PR for mixed stacking and dirty stacking are 1.27 (1.07–1.50) and 1.65 (1.19–2.25) respectively. No significant trend was observed for frequency of cooking, cooking location, and duration of cooking. Among smoke exposure indicators in the neighbourhood, only waste burning frequency in the neighbourhood and living close to a major road were associated with the risk of anemia. Frequency of waste burning in the neighbourhood decreased with decreasing anemia risk in an exposure-response manner (*p* = 0.0207). Burning garbage in the neighbourhood almost daily and burning garbage in the neighbourhood at least once a month versus not burning garbage were 1.23 (1.02–1.60) and 1.15 (0.99–1.26) respectively. Living close to major road was marginally associated with the risk of anemia (1.08, 0.97–1.40). Of the indicators of smoke exposure at the workplace, being a fish smoker was the only factor associated with the risk of anemia (1.22, 0.99–1.40), but the effect estimate was significant at borderline.

### Association of smoke exposure indicators with hemoglobin levels

The results of the association between smoke exposure indicators and hemoglobin levels are shown in Table 1. Burning different types of waste simultaneously in the household was associated with a decrease in HB in an exposure-response manner, such that burning 1 or 2 different types of waste at the same time in the neighbourhood was associated with marginal reduction (regression coefficient (β): -0.01, -0.97- -0.99) whereas, burning more than 3 different types of waste simultaneously resulted in a greater decline (-0.14, -0.77- -0.05) in HB. Current smoking behaviour was also associated with HB level in an exposure-response manner. Compared to never smokers, smoking almost daily was associated with a much higher reduction in HB (-1.40, -2.01- -0.79) than smoking at least once in a month (-1.14, -1.79- -0.48). With respect to SHS, exposure to SHS almost daily was significantly associated with HB level (β=-0.77, -1.30- -0.24). Fuel stacking in the household was also inversely associated with HB level in an exposure-response manner. The β for mixed stacking and dirty stacking compared to clean stacking were − 0.93 (-1.33- -0.21) and − 0.34 (-0.85- -0.17) respectively. Also compared to cooking 1–2 times in a month, cooking almost daily was associated with much higher decline in HB (-1.38, -2.03- -0.73) compared to cooking 1–3 times in a week (-1.04, -1.69- -0.41). Compared to cooking in an open space/shed, cooking in an enclosed space was associated with a decline in HB level (-2.04, -1.7- -0.75) but not in a combined area. Of the smoke exposure indicators in the neighbourhood, any smoke exposure indicator in the neighbourhood (-0.84, -1.43- -0.25) and living close to a major road (-0.62, -1.09- -0.16) were associated with HB level. Being involved in fish smoking was also associated with HB levels (-0.41, -0.93- -0.12).

## Discussion

### Main findings

In our cross-sectional study of the association of multiple sources of smoke exposure with the risk of anemia/HB level, the participants’ mean HB was 12.6 ± 2.1 g/dL and their anemia prevalence were 49.1%. We found an exposure-response relationships of number of different types of waste openly burnt simultaneously, fuel stacking, and frequency of garbage burning by a neighbour with anemia risk and HB level. An exposure-response relationships was also observed for current smoking behaviour, exposure to SHS, frequency of cooking, location of cooking, and duration of cooking with HB. Living close to a major road or involvement in fish smoking, and any smoke/fumes exposure indicator in the neighbourhood were also associated with HB level.

### Methodological validity

Our study has a few advantages. To the best of our knowledge, this is the first study to investigate the relationship between various sources of smoke commonly encountered in urban informal settlements and the risk of anemia and mean HB level. Confounding factors were determined based on their statistical significance (*p* < 0.05) in relation to anemia/HB and were consistent with the literature [3, 6, 7, 9]. Data on HB/anemia were collected objectively by phlebotomists with more than ten years of laboratory experience. Participants were interviewed prior to blood sampling, and their anemia status had no bearing on their responses to the questionnaire questions. Again, the phlebotomists were unaware of the participants’ exposure status. As a result, information bias related to anemia/smoke exposure was highly unlikely. When interpreting the findings of this study, a few limitations must be considered. The study was carried out at the height of the COVID-19 pandemic when social gatherings and house-to-house visits were restricted. As a result, patients who required emergency hospital care and were at the hospital during our data collection were more likely to have been enrolled. Thus, this population was unlikely to represent the source population in the study area, limiting the generalizability of our findings. Again, our current study did not quantify particulate matter and carbon monoxide levels; instead, it relied on proxy measures derived from a questionnaire, such as fuel use, proximity to a main road, and so on, to measure smoke exposure. The main disadvantage of our exposure definition is that individuals exposed to high or low exposure levels belong to the same exposure group, which may result in exposure misclassification. However, this misclassification is likely to be non-differential and may attenuate the effect size.Furthermore, the cross-sectional design nature of our study eliminates any temporality. separating persons exposed or not exposed to smoke from open burning in this setting may be difficult. However, our questions allowed us to identify participants who were exposed to smoke from open burning (initiated by them or someone else with whom they share a space) in their own compound (a cluster of families sharing the same area) and those exposed to smoke from open burning from a neighbouring compound. This was predicated on the notion that the closer one is to the source of smoke, the higher the exposure level. Thus, in our study, those who indicated that they and their neighbours do not burn waste were considered our reference group (due to their proximity to the source of smoke). Whereas those who either participated in trash burning or whose neighbours participated in trash burning or both were considered our exposed group. The ideal approach would have been to monitor exposure levels [7]. We also did not account for other potential confounders like nutrition, wealth index, maternal history of anemia, liver diseases, Inflammatory bowel disease, gastrointestinal cancers, including colon cancer and other chronic diseases because we did not collect data on them and the may bias our effect estimates. Some of our analyses have insufficient power to detect any association, increasing the margin of error and compromising the precision of our parameter estimates.

### Comparison of our findings with previous studies

Our systematic search of the literature yielded 16 studies linking PM/air pollution/biomass fuel smoke to anemia risk/HB level in developing countries, plus three more from China, South Korea, and the USA. The risk of anemia has been documented in some, but not all, previously published studies. Mishra and Retherford [9] analyzed secondary data from the 1998-99 national family health survey (NHFS-2) in India, which collected information on height, weight, and blood HB of 29 768 children aged 0–35 months from 92 486 households. Mishra and Retherford [9] categorized households into two groups depending on whether they used clean fuel exclusively (i.e., LPG or kerosene or electricity), mixed fuel (i.e., users of both clean and dirty fuel), or biomass fuel alone. After adjusting for potential confounders, users of biomass fuel alone (relative risk ratio (RRR) = 1.84, 95% CI 1.44–2.36 = 1.88) and users of mixed fuel (RRR = 1.44, 1.22–1.94) showed a higher risk of moderate-to-severe anemia than users of clean fuel. In a follow-up study, Baranwal et al. [10] examined the NHFS-3 and, with a larger sample size (i.e., 52,868), demonstrated that using clean fuel (LPG, electricity, or biogas) significantly lowers the risk of anemia in children under the age of five years. Studies of expectant mothers [11, 13, 14], preschool aged-children [15–18], and housewives in South Korea [19], as well as secondary data analysis of the Demographic and Health Surveys for 29 countries [17], have confirmed these findings. Machisa et al. [7], on the other hand, were unable to replicate the same findings in Swazi pre-schoolers. Mean HB levels were not related to smoke exposure among communities living in the Guatemalan highlands [3]. In contrast to the findings of other studies (such as [18, 19]), but in line with prior findings (such as [9]), our investigation on fuel stacking found an exposure-response association with anemia risk. Our definition of biomass fuel smoke exposure agreed with Mishra and Retherford’s [9] definition. The authors of Baranwal et al. [10] and Amadu et al. [16] employed both clean and unclean cooking, nevertheless. LPG, biogas, kerosene, and electricity were deemed to be “clean” for cooking, whereas coal, charcoal, and other filthy fuels were deemed to be “unclean”. While Kyu et al. [17] employed moderate and high exposure to biomass fuel smoke, there was no clear explanation of this term. Fuel stacking, a common practice in households in developing countries, was not considered by any of the latter definitions, which may have biased their observed effect. In contrast with populations in India’s young children [10], sub-Saharan Africa’s adult populations [16] and Ghana’s central and Volta regions [6], our informal settlement population experienced an increased prevalence of anemia. Only indoor sources such as smoke from burning biomass fuel were considered in prior studies that related the risk of anemia to smoke exposure. Armo-Annor et al. [6] discovered that women who smoke fish outdoors in partially enclosed smokehouses have an increased risk of anemia (1.8, 1.1-3.0). Exposure to ambient PM2.5 levels among Peruvian children was significantly associated with decreased average hemoglobin levels and moderate/severe anemia [15]. These results were validated in an adult Chinese population exposed to ambient PM10, PM2.5, PM1, and NO2 as well as in an adult American population [20]. The current study identified several smoke sources, including those in households, neighbourhoods, and workplaces, which are characteristic in informal settlements. In contrast to our findings on the frequency of cooking, Armo-Annor et al. [6] observed no association between the number of days spent smoking fish and the risk of anemia. Unlike fish smoking, cooking is an everyday practice performed by households and may reflect intensity of smoke exposure. Cooking in an enclosed environment was associated with reduced average HB levels than cooking in an open area or outdoors, but the effect estimate was inconsistent.

Additionally, we demonstrated for the first time an association between risk of anemia/mean HB levels and garbage burning in the household and in the neighbourhood. Our research also indicated a link between the risk of anemia/mean HB levels and living near a busy road. The latter result is in line with earlier research on outdoor particulate matter exposure [21]. Prior studies have frequently concentrated on populations in rural communities, the general population, or urban populations. This study is the first to provide evidence of the association between various sources of smoke exposure and the risk of anemia in informal settlements. Our findings have significant public health implications for those living in informal settlements, where exposure to smoke from varieties of community sources is a common scene. It is anticipated that by 2050, the number of people living in informal settlements will double [22], along with the sources and sinks of air pollution. Governments in developing nations should put in place pragmatic measures to control smoke emissions from different sources to protect human health and well-being.

## Conclusions

Our study showed significant positive associations of number of different types of waste burnt simultaneously, location where cooking was done, and closeness to a major road with anemia risk and mean HB levels. We also noted associations of current cigarette smoking behaviour, exposure to SHS, fish smoking, and any smoke indicator in the neighbourhood with mean HB levels.

## Data Availability

The data used for the analysis is not publicly available due to privacy and confidentiality agreement but will be available by the corresponding author without undue reservation.

## Abbreviations

COHb: carboxyhemoglobin
COVID-19: Coronavirus 2019
HB: Haemogloblin
HIV: Human Immune Virus
JHS/SSS: Junior High School/Senior Secondary School
LMIC: Low Middle Income Country
LPG: Liquefied Petroleum Gas
NHFS: National Family Health Survey
PAH: Polycyclic aromatic hydrocarbons
PM: Particulate matter
PR: Prevalence Ration
RDT: Rapid Diagnostic Test
RRR: Relative Risk Ratio
SHS: Second Hand Smoke
